# Reference gene selection in bovine caruncular epithelial cells under pregnancy-associated hormones exposure

**DOI:** 10.1038/s41598-022-17069-3

**Published:** 2022-07-26

**Authors:** Magdalena Sozoniuk, Monika Jamioł, Marta Kankofer, Krzysztof Kowalczyk

**Affiliations:** 1 Institute of Plant Genetics, Breeding and Biotechnology, University of Life Sciences in Lublin, Akademicka Street 15, 20-950 Lublin, Poland; 2 Department of Biochemistry, Faculty of Veterinary Medicine, University of Life Science in Lublin, Akademicka Street 12, 20-033 Lublin, Poland

**Keywords:** Biochemistry, Biotechnology, Genetics, Molecular biology, Zoology

## Abstract

Examination of transcriptional regulation occurring during pregnancy establishment and maintenance requires the identification of endogenous reference genes characterized by high expression stability. Since the expression of some reference genes may be modulated by pregnancy-associated hormones, the goal of our study was to identify suitable reference genes unaffected by hormonal treatment. In our study bovine caruncular epithelial cells were subjected to progesterone, estrogen and prostaglandin F_2α_ treatment. Ten candidate reference genes (*ACTR1A, CNOT11, HDAC1, HPRT1, RPL19, RPS9, SDHA, SUZ12, UXT* and *ZNF131*) were evaluated with the use of four approaches (geNorm, NormFinder, BestKeeper, delta Ct). We found that *RPS9* and *SUZ12* displayed the highest expression stability in the tested material. Moreover, *HPRT1* and *SDHA* were found inappropriate for RT-qPCR data normalization as they demonstrated the highest expression variability out of all candidates analysed. Hence geNorm calculations shown that the use of just two best-performing genes would be sufficient for obtaining reliable results, we propose that *RPS9* and *SUZ12* be used as suitable endogenous controls in future studies investigating gene expression in normal and compromised pregnancies.

## Introduction

The functioning of the placenta undergo complex regulation, in which multiple hormones of different structure play an essential role. The major placental steroid hormones are progesterone (P4) and estrogen (E2). P4 is the most important hormone for a successful pregnancy, preventing myometrial contractions^1^. Estrogens, which are signals for maternal recognition of pregnancy, stimulate the growth of the endometrium and myometrium^2^. Significant body of evidence confirms not only steroid hormones but also prostaglandins involvement in gene expression regulation in the bovine uterine tissues^3–7^. Bovine uterine endometrium regulates ovarian function at the end of the oestrous cycle by secreting the non-steroidal hormone prostaglandin F_2α_ (PGF_2α_)^8^. The main role of PGF_2α_ is to trigger luteolysis and initiate parturition in cows^9^.

One of the most frequently used techniques for the gene expression analysis is quantitative reverse transcription real-time PCR (RT-qPCR), in which the expression of target genes is normalized against internal control genes called reference genes (RGs). RGs should be characterized by stable expression in tested material under given experimental conditions. Since universal endogenous reference does not exist, the selection of optimal RGs has become a fundamental step in RT-qPCR experiments^10^. Several previous studies aimed at identifying best performing RGs in bovine maternal reproductive tissues^11–14^, however, neither of them investigated the effect of steroid hormones and PGF_2α_ on RGs stability.

The uterus undergoes tissue remodeling in response to hormonal stimuli at different times of the oestrous cycle and during pregnancy. As oestrous cycle and pregnancy have a significant effect on genes expression^8,15^, the alternations in RGs stability in reproductive tissues might be expected. The study of Berruien et al. found that pregnancy influenced the RGs selection in murine tissues^16^. Other studies reported that the presence of sex hormones either upregulated or downregulated the expression of several commonly used RGs in mouse uterus^17,18^ and in bovine corpus luteum^11^. This highlights the need of determining which RGs are characterized by unaltered expression under various hormonal exposure.

Although limited studies examined blood transcriptome profiles of cows experiencing miscarriage^19,20^, transcriptional changes occurring in the bovine reproductive tissues leading to embryonic mortality and/or fetal loss are yet to be elucidated. Such studies are crucial for the identification of genes and pathways involved in healthy pregnancy maintenance. Identifying suitable endogenous references stably expressed under various hormonal treatments will facilitate further RT-qPCR studies providing additional insight in this area. Therefore, our study aimed to evaluate RGs stability in primary placental cell cultures obtained from bovine pregnant caruncles under steroid hormones and PGF_2α_ exposure.

## Results

### Efficiency and specificity of amplification

A set of ten potential reference genes (*ACTR1A*, *CNOT11*, *HDAC1, HPRT1, RPL19, RPS9, SDHA, SUZ12, UXT, ZNF131*) was assessed in terms of their expression stability in the tested material. Prior to this, primer specificity was evaluated which is crucial for obtaining correct results. Primer pairs (either obtained from literature or designed within this study) consistently generated single amplicons, as confirmed by dissociation curves analysis (Fig. S1). Primers efficiencies ranged from 90.6% (*ZNF131*) to 107% (*RPS9*), with all regression coefficients ≥ 0.990. The quantification cycle values (C_q_) oscillated between 15.05 and 28.54 (Fig. S2). Amplification details are shown in Table S1.

### Expression stability analysis by geNorm

**Figure 1:**
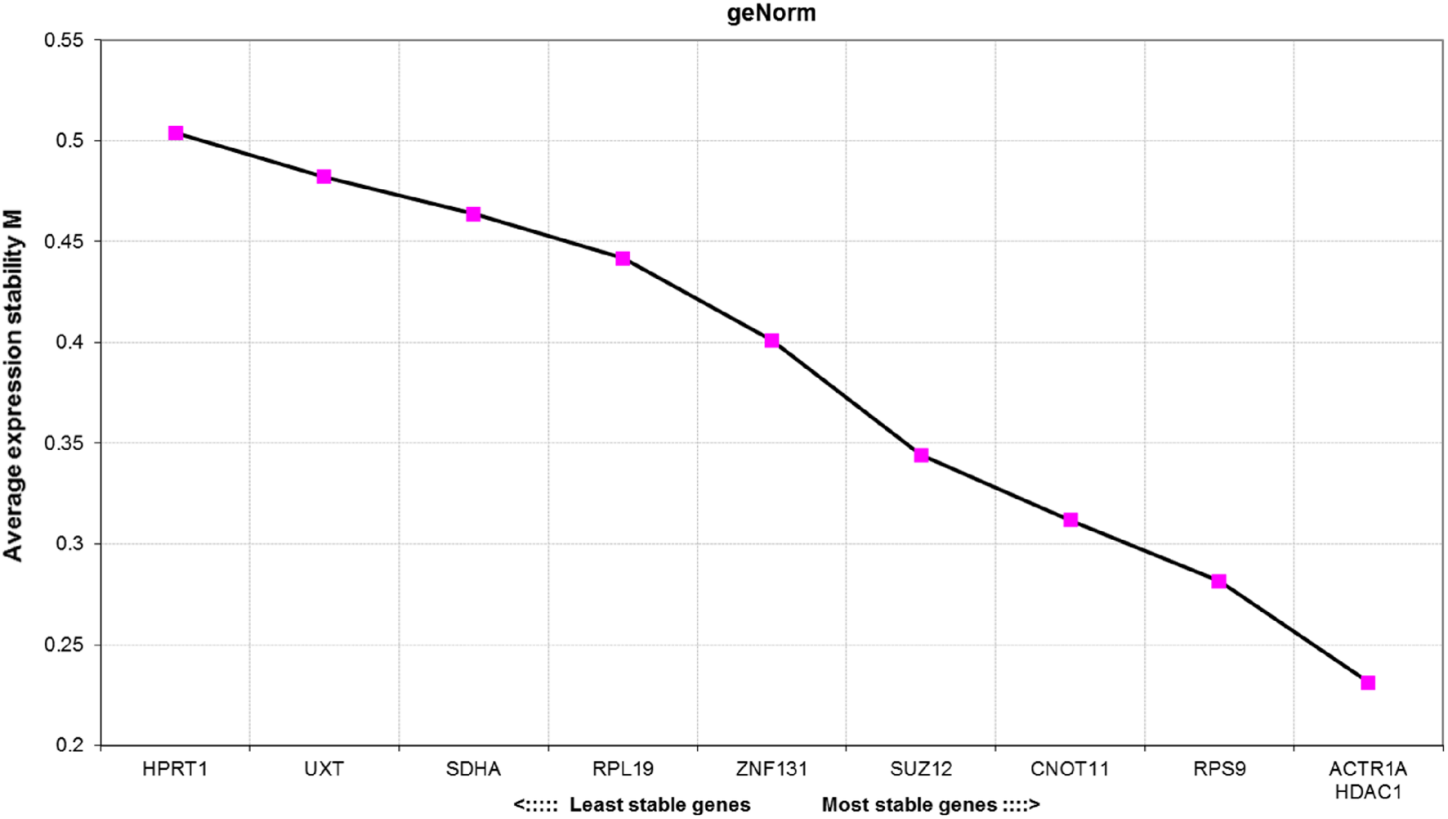
Average expression stability M of candidate reference genes calculated by the geNorm algorithm. The lower the M value, the more stably is the gene expressed in the tested samples.

The ranking generated by the geNorm algorithm is based on average expression stability values (M), with RGs which have the lowest M values being considered the most stable ones. According to the obtained results (Fig. 1), the best performing genes were *ACTR1A* and *HDAC1* (M = 0.231). High expression stability in tested samples was also displayed by *RPS9*, *CNOT11* and *SUZ12*. On the other hand, *HPRT1* and *UXT* were characterized by the highest expression variability.

### Expression stability analysis by NormFinder

**Figure 2:**
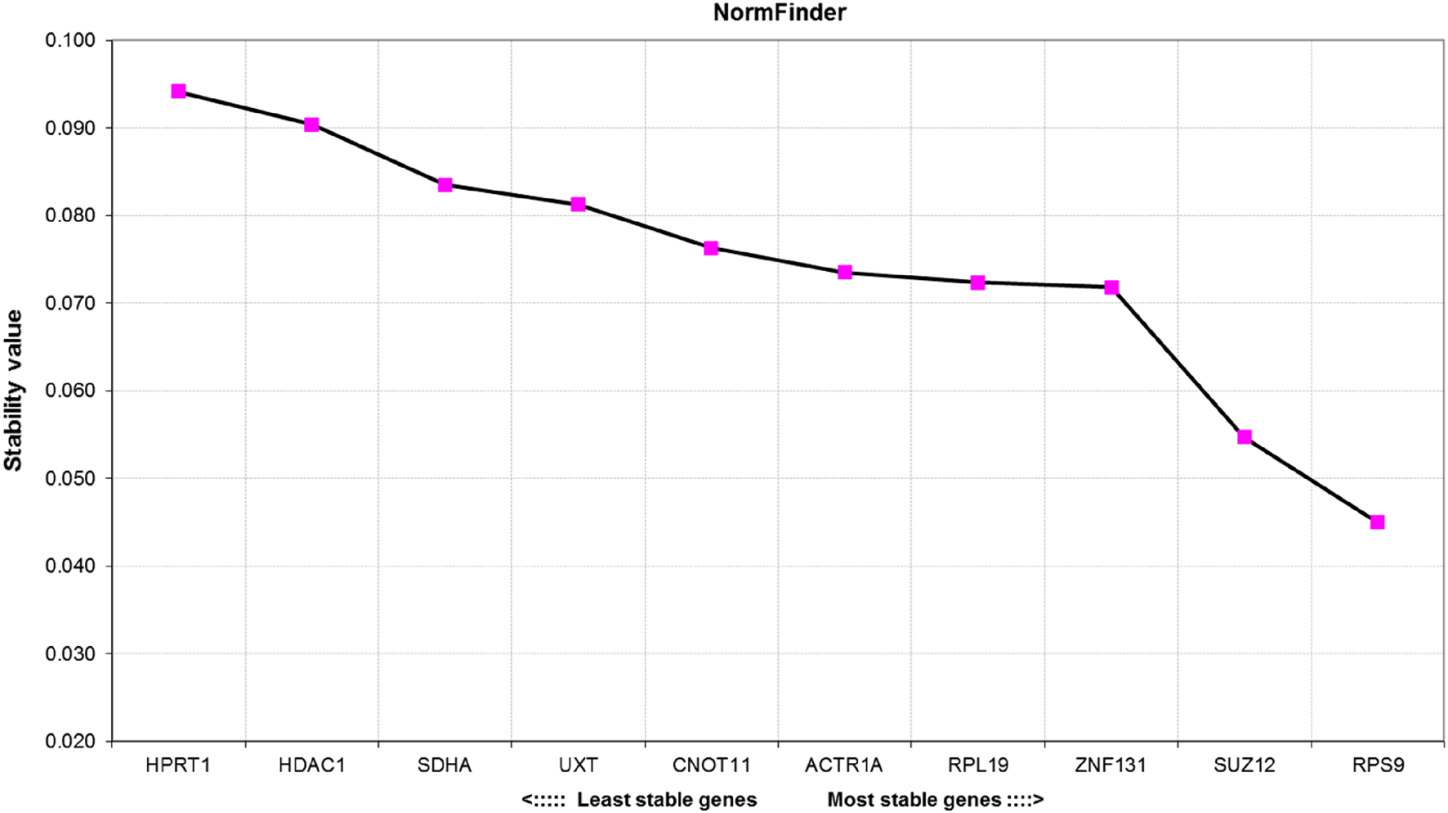
Stability value (SV) of candidate reference genes calculated by the NormFinder algorithm. The lower the SV, the more stably is the gene expressed in the tested samples.

The NormFinder algorithm calculates a stability value (SV) for each candidate RG which is based on both intra-group and inter-group variation. The stability values (SV) of all tested RGs assessed by the NormFinder algorithm are shown in Fig. 2. Genes with low inter- and intra-group variation within tested material are characterized by low SV and therefore considered as best candidates for RT-qPCR data normalization. Here, *RPS9* (SV = 0.045) and *SUZ12* (SV = 0.055) were found to be the most stable, whereas *HPRT1* and *HDAC1* exhibited the highest expression variation.

### Expression stability analysis by BestKeeper

**Figure 3:**
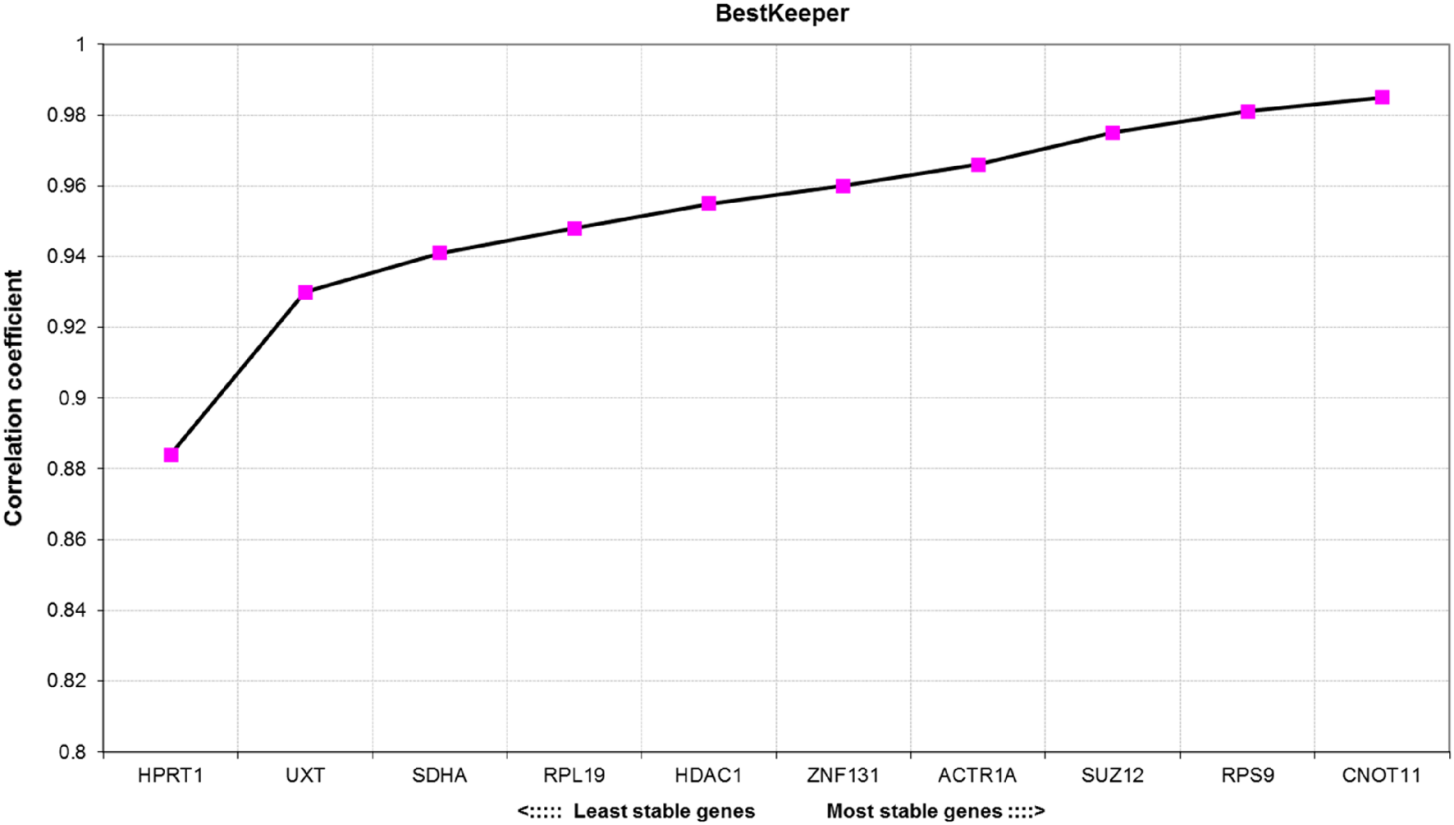
Correlation coefficient (*r*) of candidate reference genes calculated by the BestKeeper algorithm. The higher the correlation coefficient, the more stably is the gene expressed in the tested samples.

The BestKeeper calculates descriptive statistics of the Cq values, among which the main parameters used in the evaluation of potential RGs are Pearson correlation coefficient of each individual gene with the geometric mean of all genes (the BestKeeper Index) and the standard deviation (SD). The most stably expressed genes are those exhibiting the highest coefficient of correlation (the closer to 1 the better). Moreover, any candidate gene with SD higher than 1 is considered unsuitable for expression data normalization and should be excluded from further analysis^21^. Here, all tested RGs displayed SD below 1. As shown in Fig. 3, *CNOT11* and *RPS9* were selected by BestKeeper as best scoring RGs, since their correlation coefficient (*r*) was most closely related to 1 (*r* = 0.985 or *r* = 0.981, respectively). Nevertheless, a strong correlation (with *r* > 0.9) was noticed for all tested RGs except for *HPRT1*.

### Expression stability analysis by delta Ct method

**Figure 4:**
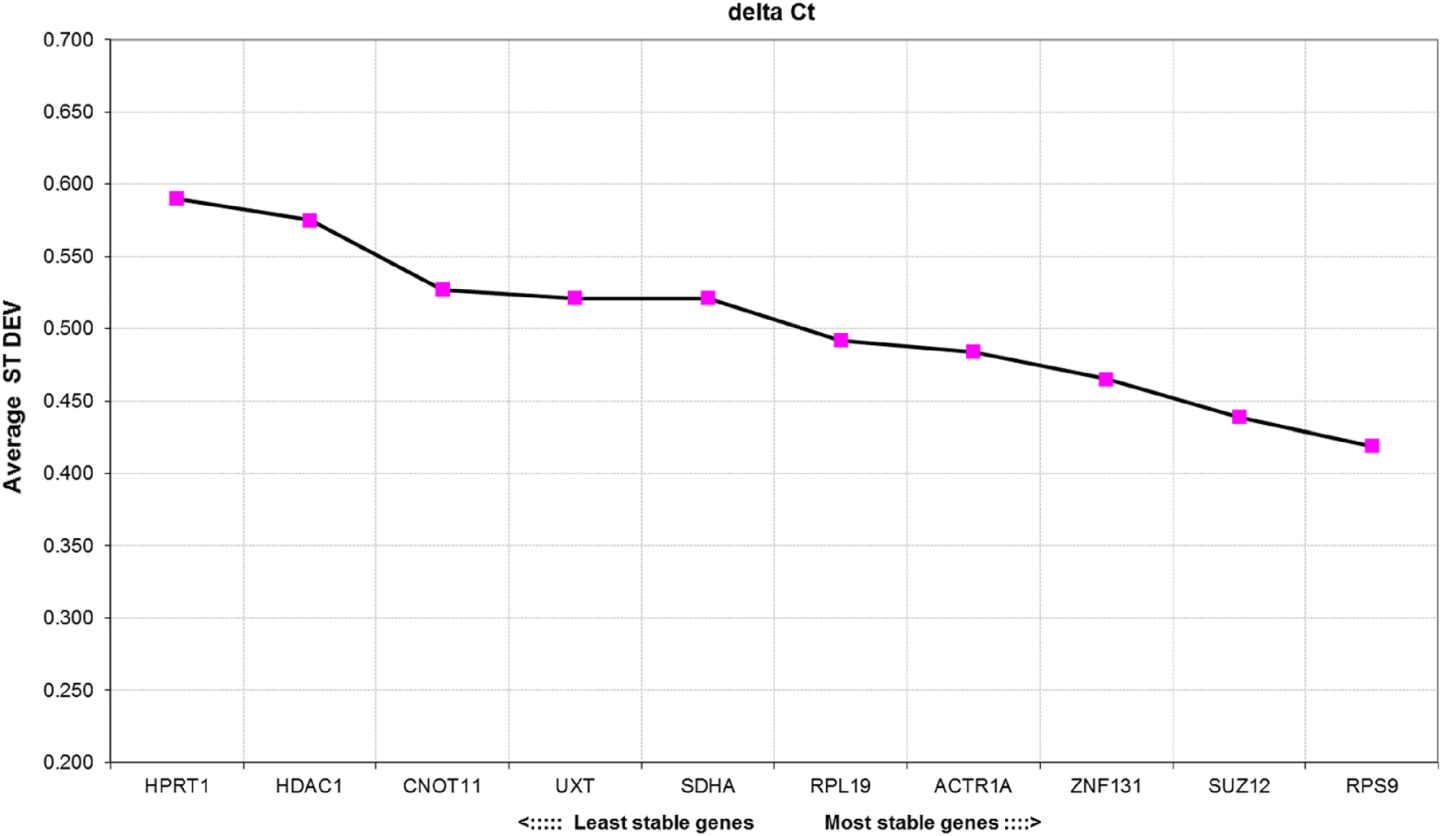
The average standard deviation of candidate reference genes calculated by the delta Ct method. The lower the mean ST DEV, the more stably is the gene expressed in the tested samples.

According to the delta Ct method *RPS9* (SD = 0.419) and *SUZ12* (SD = 0.439) were the least variable genes out of all RGs tested (Fig. 4). *ZNF131*, *ACTR1A* and *RPL19* also performed well, which is reflected in their low mean SD (< 0.5). *HPRT1* and *HDAC1* were found to be the least stable in the tested material, as they were characterized by the highest mean SD.

### Determination of best RGs for RT-qPCR data normalization

**Figure 5:**
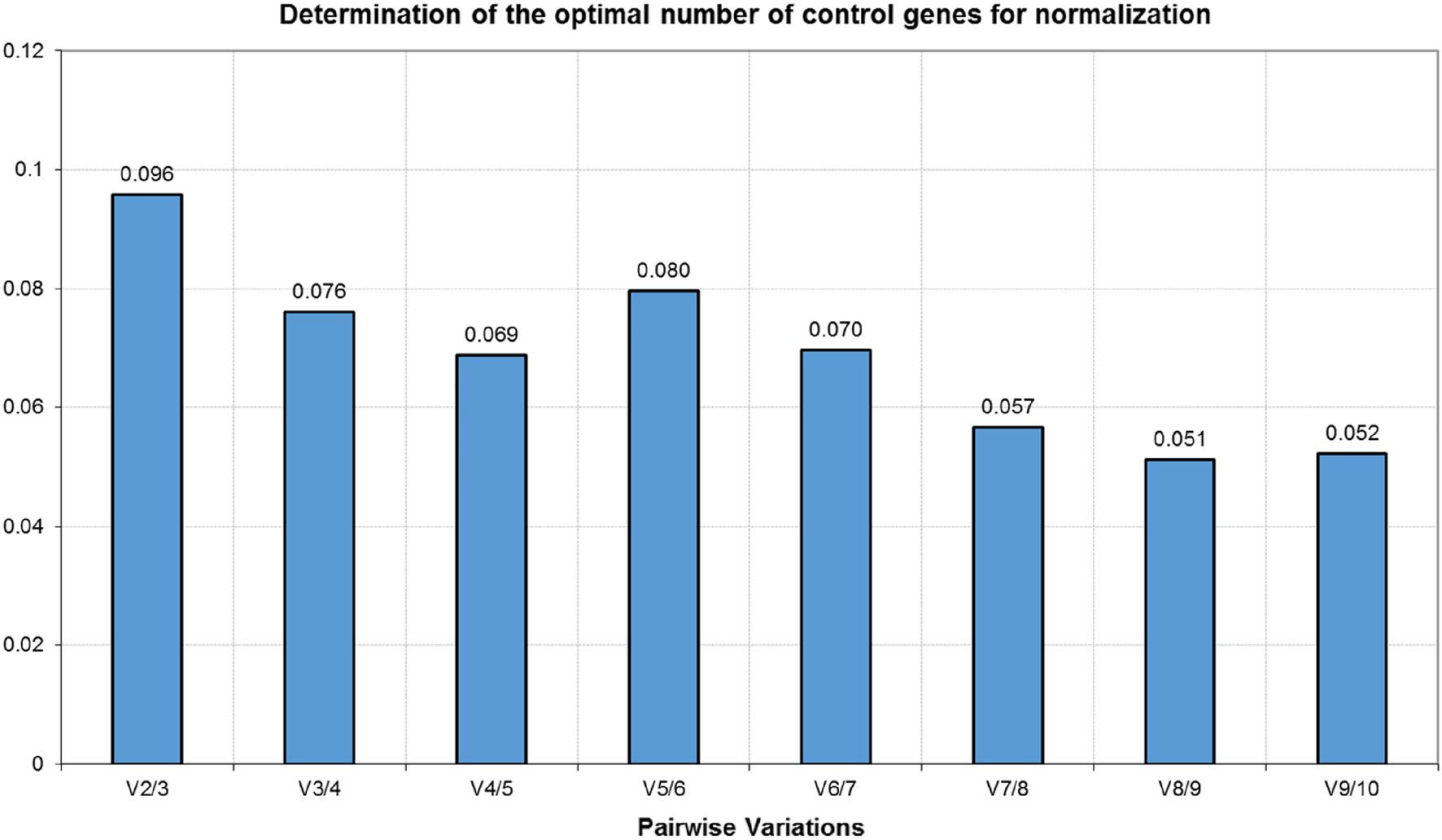
Determination of the optimal number of reference genes calculated by the geNorm algorithm. Pairwise variation (V_*n*_/V_*n*+*1*_) lower than 0.15 indicates no significant contribution made by the inclusion of an additional reference gene.

One of the additional features of the geNorm algorithm is that it allows determining the optimal number (*n*) of RGs for RT-qPCR data analysis. Calculated pairwise variation (V_*n*_/V_*n*+*1*_) of below 0.15 indicates that the *n* number of RGs is sufficient for obtaining reliable results, hence the inclusion of *n* + *1* RG is not necessary and would not make any significant contribution to the analysis. In the present study, the V_*2*/*3*_ equalled 0.096 (Fig. 5) suggesting that the use of just two best performing RGs would guarantee proper data normalization.

The summary of obtained results, together with the comprehensive ranking of tested RGs, is presented in Table 1. Due to the different calculation methods, the order of RGs varies between the algorithms. However, the best performing RGs proposed by NormFinder (*RPS9* and *SUZ12*) are consistent with those indicated by the delta Ct method. Moreover, they are among the top three RGs pointed out by BestKeeper and are shown to have high/good expression stability by geNorm. Furthermore, irrespective of the calculation method *HPRT1* ranked in the last position, suggesting its highest expression variability among tested genes. From the overall final ranking, *RPS9* and *SUZ12* are proposed as the best pair of RGs for RT-qPCR data normalization in the tested material.

**Table 1:** The comprehensive ranking of candidate reference genes stability according to all tested algorithms. The ranking was generated using the geometric mean of the ranks.

Rank		geNorm		NormFinder		BestKeeper		Delta Ct		Comprehensive ranking
		Gene	M	Gene	SV	Gene	r	Gene	Average SD	
Best	1	*ACTR1A* *HDAC1*	0.231	*RPS9*	0.045	*CNOT11*	0.985	*RPS9*	0.419	*RPS9*
	2	–	–	*SUZ12*	0.055	*RPS9*	0.981	*SUZ12*	0.439	*SUZ12*
	3	*RPS9*	0.281	*ZNF131*	0.072	*SUZ12*	0.975	*ZNF131*	0.465	*ACTR1A*
Good	4	*CNOT11*	0.312	*RPL19*	0.072	*ACTR1A*	0.966	*ACTR1A*	0.484	*CNOT11*
	5	*SUZ12*	0.344	*ACTR1A*	0.074	*ZNF131*	0.960	*RPL19*	0.492	*ZNF131*
	6	*ZNF131*	0.401	*CNOT11*	0.076	*HDAC1*	0.955	*UXT*	0.521	*HDAC1*
Average	7	*RPL19*	0.442	*UXT*	0.081	*RPL19*	0.948	*SDHA*	0.521	*RPL19*
	8	*SDHA*	0.464	*SDHA*	0.083	*SDHA*	0.941	*CNOT11*	0.527	*UXT*
	9	*UXT*	0.482	*HDAC1*	0.090	*UXT*	0.930	*HDAC1*	0.575	*SDHA*
	10	*HPRT1*	0.504	*HPRT1*	0.094	*HPRT1*	0.884	*HPRT1*	0.590	*HPRT1*

In order to validate the suitability of selected RGs, expression analysis of *PGR* was performed. Normalization was conducted either with two top RGs (*RPS9* + *SUZ12)* or the least stable RG, *HPRT1* (Figure S3). When *HPRT1* was used as internal control, changes in expression profiles could be observed in E2- and PGF_2α_-treated samples, leading to overestimation of *PGR* transcript level.

## Discussion

The RT-qPCR data normalization performed against endogenous reference affected by experimental treatment or tissue physiological status may greatly alter interpretation of the results and lead to inaccurate and/or erroneous conclusions^16^. Since steroid hormones were found to modulate the transcript levels of some of the traditionally used RGs (e.g. *GAPDH* and *18s RNA* transcription altered by estrogen and progesterone, respectively)^11,18^ identification of RGs unaffected by hormonal changes is required.

In our study, we evaluated the expression stability of 10 candidate RGs in placental cell cultures obtained from bovine caruncles during pregnancy. The cell cultures were subjected to a hormonal treatment of P4, E2 and PGF_2α_. Stability rankings generated with the use of four algorithms (geNorm, NormFinder, BestKeeper and delta Ct method) revealed that *RPS9* and *SUZ12* displayed the highest expression stability in the tested material. Furthermore, according to geNorm calculations, only two best performing RGs suffice to appropriately normalize RT-qPCR data. Our study also found that *HPRT1* and *SDHA* were characterized by the highest variability out of all RGs analysed.

While studying maternal-conceptus immune tolerance in bovine endometrium Chaney et al. assayed a set of eight potential reference genes in terms of their expression stability^22^. For caruncular tissues treated with either progesterone (P4), recombinant bovine galectin-1 (rbLGALS1) or their combination, ring finger protein 11 gene (*RNF11*) and H3 histone family member 3A gene (*H3F3A*) were chosen as internal controls. Similarly to our results, *SDHA* gene was not among the most stable RGs in caruncular endometrium. Nevertheless, it proved to be among the two-best RGs stably expressed in intercaruncular bovine endometrium treated with rbLGALS1.

Likewise, *SDHA* along with *PPIA* (peptidylprolyl isomerase A gene) were identified as the most stable RGs across bovine endometrial explants subjected to various treatments (e.g. co-cultured with bovine conceptuses of different origin or exposed to interferon tau (IFNT)^13^. Moreover, *SDHA* was proposed as a good internal control in studies performed on the uterus of sows across pregnancy^23^. In the research on placental formation in cows carried out by Guillomot et al.^24^ *SDHA*, *RPL19* and *RPLP0* were chosen for the normalization of RT-qPCR data obtained from placental tissues, endometrial caruncles and the intercaruncular endometrium. By contrast, *SDHA* was demonstrated to display high expression variability in the uterus of both non-pregnant and pregnant mice^16^.

The primary function of the corpus luteum is the secretion of progesterone, which prepares the endometrium for implantation and it is essential for the maintenance of pregnancy^12^. The research of Rekawiecki et al. performed on corpora lutea collected from cyclic or pregnant cows revealed high expression stability of *SUZ12* and *CNOT11* (alternatively named *C2orf29*)^11^. This is in accordance with our results which also demonstrated high expression stability of *SUZ12* and rather good expression stability of *CNOT11*.

In a study carried out by Mezera et al. in the bovine corpus luteum throughout early pregnancy and luteolysis a set of 74 stably expressed genes was identified, one-third of which belonged to the ribosomal protein family^25^. As protein synthesis is an essential process for the cell, nearly constant expression of these genes might be expected. However, the abovementioned study revealed also that *RPS4X* (ribosomal protein S4X) and *RPL4* (ribosomal protein L4) may be used as internal controls as long as the samples were collected throughout luteolysis. In specimens coming from early pregnancy other candidates performed much better, therefore they should be chosen for data normalization. This constitutes yet another example that corroborates the need for putative RGs assessment in specific samples sets. Still, research carried out in other bovine cells and tissues confirms the potential of ribosomal protein subunit genes to serve as internal controls in RT-qPCR data analysis^26–28^.

Due to the limited research performed on bovine species, a short reference to the results obtained by other Authors in some non-bovine species was also made. Regarding the prospective use of genes encoding ribosomal protein subunits as RGs, worth mentioning is that *RPL13a* (ribosomal protein L13a) expression was found not to be altered by estrogen treatment in the murine uterus^18^. In relation to hormone-secreting organs, ribosomal genes *RPS9* and *RPS18* were identified as the best RGs in bovine ovaries, regardless of their luteal stages^12^.

Cheng et al. performed RGs selection within complex samples set of fetal tissues and maternal reproductive tissues obtained from early-pregnant cows^14^. The analysis of eight tissue types was carried out, including caruncular endometrium samples. According to obtained data, which was analysed by three algorithms (geNorm, NormFinder and BestKeeper), *RPS9* and *CNOT11* displayed the highest expression stability across all of the examined tissues. Likewise, a comprehensive ranking generated within this study points out to *RPS9* as the most stable gene, whereas *CNOT11* is reported to have good expression stability. In the aforementioned study, *GAPDH* and *HPRT1* were indicated as the least stable RGs by all used algorithms. This is also in concordance with our results, which ranked *HPRT1* as the worst-performing gene out of all candidates tested. However, we did not test *GAPDH*. On the other hand, some studies identified *HPRT1* as one of the two best RGs in the porcine placenta and ovaries but not in the porcine endometria^23,29,30^.

As stated by Craythorn et al., the identification of appropriate RGs in uterine tissues might be a challenge due to the structural alternations occurring in response to circulating hormones^17^. The literature shows that both *GAPDH* and *HPRT1* expression is modulated by estrogens in the mouse uterus^18^. Furthermore, their expression was reported to be altered by sex hormone exposure in human skin cells^31^.

Selection of RGs conducted in ovaries of riverine buffaloes (*Bubalus bubalis*) showed that *GAPDH* displayed the most variable expression out of 10 candidates analysed^32^. Similarly, *GAPDH* ranked as the least stable RG in the corpus luteum collected from cows in the first 2 months of pregnancy^25^. Moreover, *GAPDH* and *HPRT1* (along with *SDHA*) belonged to the least stably expressed RGs in bovine ovaries^12^. Nonetheless, *GAPDH* continues to be used as the internal control in many gene expression studies of bovine reproductive tissues^33–36^.

Summarizing, based on the recommendations of many studies, the gene expression results are more reliable as they are normalized by geometric means of multiple reference genes^32,37,38^. Moreover, the literature shows that RGs selected in some tissues under certain conditions are not necessarily the best performing genes under other circumstances. Our present data has demonstrated that two reference genes should be sufficient to validate expression data across bovine caruncular epithelial cells. In conclusion, *RPS9* and *SUZ12* were most stable and appropriate reference genes identified in this study and their geometric means would provide accurate normalization factor for expression data in epithelial cells derived from maternal part of bovine placenta. Therefore, based on the results of this study, we propose *RPS9* and *SUZ12* to be used as the best candidates for internal controls in expression experiments carried out on bovine caruncular epithelial cell cultures exposed to pregnancy-associated hormones, like P4, E2 and PGF_2α_. Our findings have important implications for future studies, as transcriptional regulation of the establishment and maintenance of pregnancy is still poorly understood.

## Materials and methods

### Primary cell cultures

Four primary placental cell cultures were obtained from bovine pregnant caruncles after slaughter (2nd month of pregnancy: n = 2; 4th month of pregnancy: n = 2) as described previously^39^. The cells (0.5 × 10^6^ cells/well) were seeded in six-well plates in a 2 ml full-supplemented medium containing Dulbecco's Modified Eagle Medium/Nutrient Mixture F-12 (DMEM/F-12) 50:50 (15–090-CVR, Corning), 10% fetal bovine serum (FBS) (35-079-CV, Corning), 100 IU/ml penicillin/100 μg/ml streptomycin (30-002-CI, Corning) and 2 mM l-glutamine (G7513, Sigma-Aldrich) and incubated (37 °C, 5% CO_2_) for 24 h. The culture media were then changed to the media without FBS and the cells were incubated again (37 °C, 5% CO_2_) for 24 h. Next day, the cells were exposed to 2 ml media containing 10^–12^ mol/l E2, 10^–8^ mol/l P4, 10^–10^ mol/l PGF_2α_ or PBS (control) for 24 h (37 °C, 5% CO_2_). In this way, four experimental groups were obtained in which each well was treated differently.

All experiments were performed without the use of live animals.

### mRNA extraction

After discarding the medium, the wells with cells were washed with 5 mM EDTA/PBS and the cells were then trypsinized (0.5% trypsin in 5 mM EDTA/PBS) until detached. The cells were then carefully resuspended in a culture medium and centrifugated (200×*g*, 5 min, RT). Obtained cell pellets were then washed with PBS and centrifuged again (200×*g*, 5 min, RT). The cell pellets were carefully dissolved in 0.5 ml lysis buffer (pH  7.5; 100 mM Tris, 500 mM LiCl, 10 mM EDTA, 1% SDS, 5 mM DTT) with 0.5 μl RNase inhibitor (EO0381, Thermo Scientific™). The mRNA isolation from the cell lysates was performed using Dynabeads^®^ Oligo (dT)_25_ (Invitrogen™) according to the manufacturer’s guidelines. The 500 μl magnetic beads were transferred to a 1.5 ml tubes and placed on a magnet (Merck Millipore PureProteome™ Magnetic Stand) for 2 min. After discarding the supernatants, the tubes were removed from the magnet, 500 μl binding buffer (pH  7.5; 20 mM Tris, 1 M LiCl, 2 mM EDTA) was added and the tubes were placed on the magnet (2 min). The incubation with binding buffer was repeated, as above. The buffer was removed and 0.5 μl lysates were added and mixed precisely with magnetic beads. The mRNA annealing was performed for 8 min (mixing, RT). The tubes were placed on the magnet (2 min) and then the supernatants were removed. The beads were thoroughly washed (totally 3 × , RT) with appropriate buffers to separate the beads with mRNA from the supernatants. Buffer A (pH  7.5; 10 mM Tris, 0.15 M LiCl, 1 mM EDTA, 0.1% SDS) was used for the first wash, followed by 2 × washing with buffer B (pH  7.5; 10 mM Tris, 0.15 M LiCl, 1 mM EDTA). Elution of mRNA was performed using 20 μl 10 mM Tris–HCl (pH  7.5) with 0.5 μl RNase inhibitor (4 min at 80 °C). The tubes were placed on the magnet and the supernatants containing the mRNA were quickly transferred to a new tube placed in ice. The concentration and purity of extracted mRNA was assessed spectrophotometrically on NanoDrop 2000 (Thermo Scientific™).

### RT-qPCR

The cDNA synthesis and DNase treatment were conducted with the use of Maxima First Strand cDNA Synthesis Kit for RT-qPCR, with dsDNase (Thermo Scientific™) following the manufacturers’ recommendations. The reverse transcription was performed in 20 µl reactions containing 0.5 µg of mRNA. In our preliminary research, a wider set of candidate genes was tested. Only primer pairs which met all amplification criteria (amplification efficiency between 90 and 110%, regression coefficients ≥ 0.990, presence of a single amplicon on the dissociation curve) were used for gene expression analyses. Ten candidate RGs chosen for further analysis are as follows: *ACTR1A* (actin-related protein 1A), *CNOT11* (CCR4-NOT transcription complex subunit 11), *HDAC1* (histone deacetylase 1)*, HPRT1* (hypoxanthine phosphoribosyltransferase 1)*, RPL19* (ribosomal protein L19)*, RPS9* (ribosomal protein S9)*, SDHA* (succinate dehydrogenase complex flavoprotein subunit A)*, SUZ12* (SUZ12 polycomb repressive complex 2 subunit)*, UXT* (ubiquitously expressed prefoldin like chaperone) and *ZNF131* (zinc finger protein 131). Primers for gene expression analyses were taken from previous studies or designed with PrimerBLAST tool^40^ as indicated in Table 2.

**Table 2:** Primer details of selected candidate reference genes and target gene.

Gene	Gene product	GenBank accession no	Primer sequences (5′–3′)	Product length (bp)	References
*ACTR1A*	Actin related protein 1A	NM_001193248.3	F: AAGTCTGACATGGACCTGCG	130	This study
			R: TCTTCACGTCTTTCGGAGCC		
*CNOT11*	CCR4-NOT transcription complex subunit 11	XM_002691150.5	F: TCAGTGGACCAAAGCCACCTA	169	Rekawiecki et al.^11^
			R: CTCCACACCGGTGCTGTTCT		
*HDAC1*	Histone deacetylase 1	NM_001037444	F: TTACGACGGGGATGTTGGAA	136	This study
			R: GGCTTTGTGAGGGCGATAGA		
*HPRT1*	Hypoxanthine phosphoribosyltransferase 1	NM_001034035.2	F: GGATTACATCAAAGCACTGAACA	193	Cheng et al.^14^
			R: CATTGTCTTCCCAGTGTCAATT		
*RPL19*	Ribosomal protein L19	NM_001040516.2	F: TCGCTGTGGCAAGAAGAAAGTCTGG	102	Schoen et al.^12^
			R: AGCCCATCTTTGATCAGCTTCCG		
*RPS9*	Ribosomal protein S9	NM 001101152.2	F: CTGAAGCTGATCGGCGAGTA	119	This study
			R: GGGTCTTTCTCATCCAGCGT		
*SDHA*	Succinate dehydrogenase complex flavoprotein subunit A	NM_174178	F: GCAGAACCTGATGCTTTGTG	185	Guillomot et al.^24^
			R: CGTAGGAGAGCGTGTGCTT		
*SUZ12*	SUZ12 polycomb repressive complex 2 subunit	NM_001205587.3	F: GAACACCTATCACACACATTCTTGT	130	Walker et al.^41^
			R: TAGAGGCGGTTGTGTCCACT		
*UXT*	Ubiquitously expressed prefoldin like chaperone	NM_001037471	F: CAGCTGGCCAAATACCTTCAA	125	Kadegowda et al.^42^
			R: GTGTCTGGGACCACTGTGTCAA		
*ZNF131*	Zinc finger protein 131	NM_001101218.1	F: AGAAAGAAGCTTTATGAATGTCAGG	94	Walker et al.^41^
			R: GTTTATCTCCAGTGTGTATCACCAG		
*PGR*	Progesterone receptor	NM_001205356.1	F: CTACCTTAGGCCGGATTCAGA	200	This study
			R: TGCAATCGTTTCTTCCAGCACAT		

The RT-qPCR reactions were conducted using PowerUp™ SYBR™ Green Master Mix (Applied Biosystems™) according to the manufacturer’s instructions. Reaction mixtures had a final volume of 20 µl and contained 2.5 ng of cDNA and 200 nM of each primer. The cycling conditions were as follows: 2 min at 50 °C, 2 min at 95 °C, 40 cycles of 15 s at 95 °C and 1 min at 60 °C. In order to verify the specificity of PCR products melting curve analysis was performed after each run with continuous data collection from 60 to 95 °C. The reactions were performed in two technical replicates along with no template control (NTC) and no reverse transcription control (RT-). Standard curves were generated from serial dilution of pooled cDNA and were used to determine amplification efficiencies for each primer pair. All qPCR reactions were conducted on QuantStudio 3 apparatus (Applied Biosystems™) and the raw data was subjected to analysis using a dedicated relative quantification software module from ThermoFisher Cloud (ThermoFisher Scientific).

### Stability analysis of candidate reference genes

The stability of candidate RGs expression was assessed using four different algorithms, that is geNorm^38^, NormFinder^43^, BestKeeper^21^ and delta Ct method^44^. Briefly, the geNorm calculates the average expression stability value (M) based on the mean pairwise variation of a particular gene with all other tested candidate genes^38^. The NormFinder estimates the stability based on the intra-group and inter-group variation^43^. The BestKeeper generates several statistical parameters, of which the coefficient of correlation (r) to the BestKeeper Index (which is the geometric mean of Cq values of all reference genes) allows to rank the genes according to their stability^21^. Finally, the delta Ct method compares the relative expression of pairs of genes within each sample to identify stable RGs^44^. Efficiency corrections were taken into consideration during stability analysis. A comprehensive ranking of candidate RGs was compiled as suggested by Velada et al.^45^ based on the geometric mean of the ranks. For the validation of RGs, expression analysis of the target gene encoding progesterone receptor (*PGR*) was carried out. RT-qPCR reaction conditions were as described above. The relative gene expression was calculated according to 2^-ΔΔCt^ method.

## Supplementary Information

Supplementary Information.

## Data Availability

The datasets used and/or analysed during the current study available from the corresponding author on reasonable request.
