# Supplementary material for: Reference gene selection in bovine caruncular epithelial cells under pregnancy-associated hormones exposure
Source: Sci Rep. 2022 Jul 26;12:12742. doi: 10.1038/s41598-022-17069-3 (PMC9325760; doi:10.1038/s41598-022-17069-3)
Supplement: Supplementary file 1 — Supplementary Information. [file 41598_2022_17069_MOESM1_ESM.pdf]

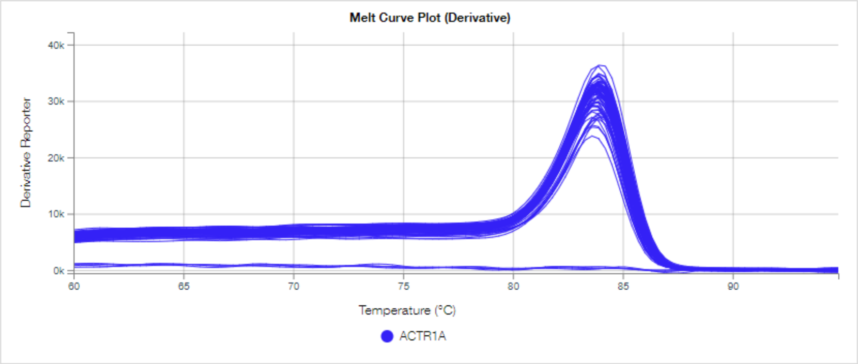
Melt Curve Plot (Derivative)
Derivative Reporter
40k
30k
20k
10k
0k
60
65
70
75
80
85
90
Temperature (°C)
ACTR1A

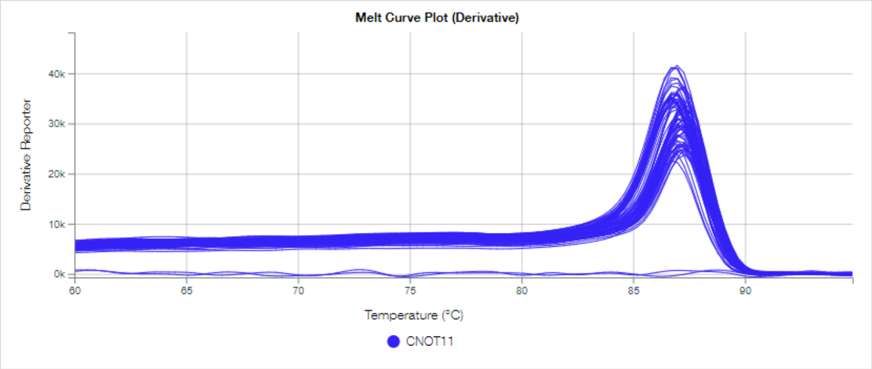
Melt Curve Plot (Derivative)
Derivative Reporter
40k
30k
20k
10k
0k
60
65
70
75
80
85
90
Temperature (°C)
CNOT11

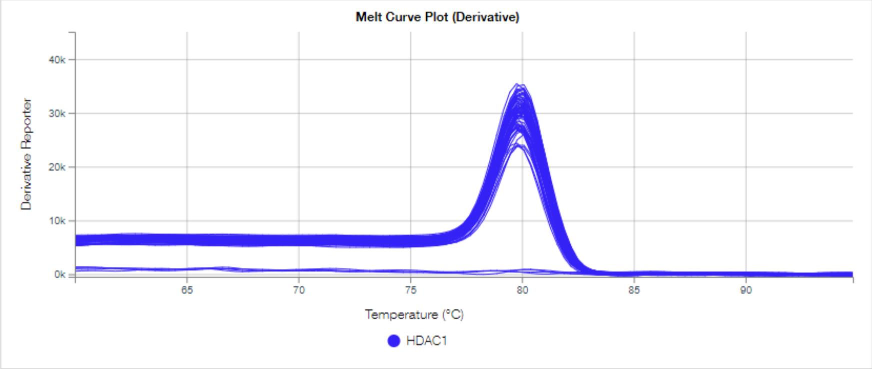
Melt Curve Plot (Derivative)
Derivative Reporter
40k
30k
20k
10k
0k
65
70
75
80
85
90
Temperature (°C)
HDAC1

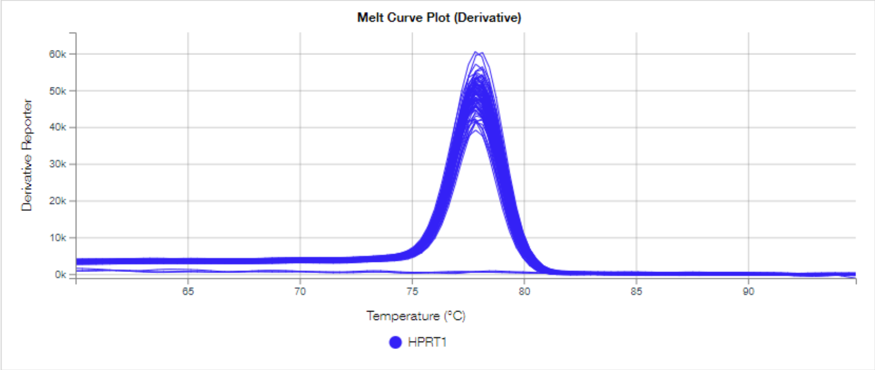
Melt Curve Plot (Derivative)
Derivative Reporter
60k
50k
40k
30k
20k
10k
0k
65
70
75
80
85
90
Temperature (°C)
HPRT1

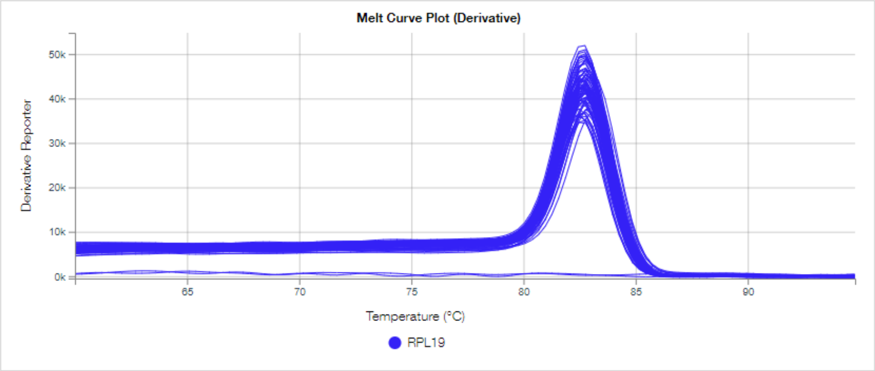
Melt Curve Plot (Derivative)
Derivative Reporter
50k
40k
30k
20k
10k
0k
65
70
75
80
85
90
Temperature (°C)
RPL19

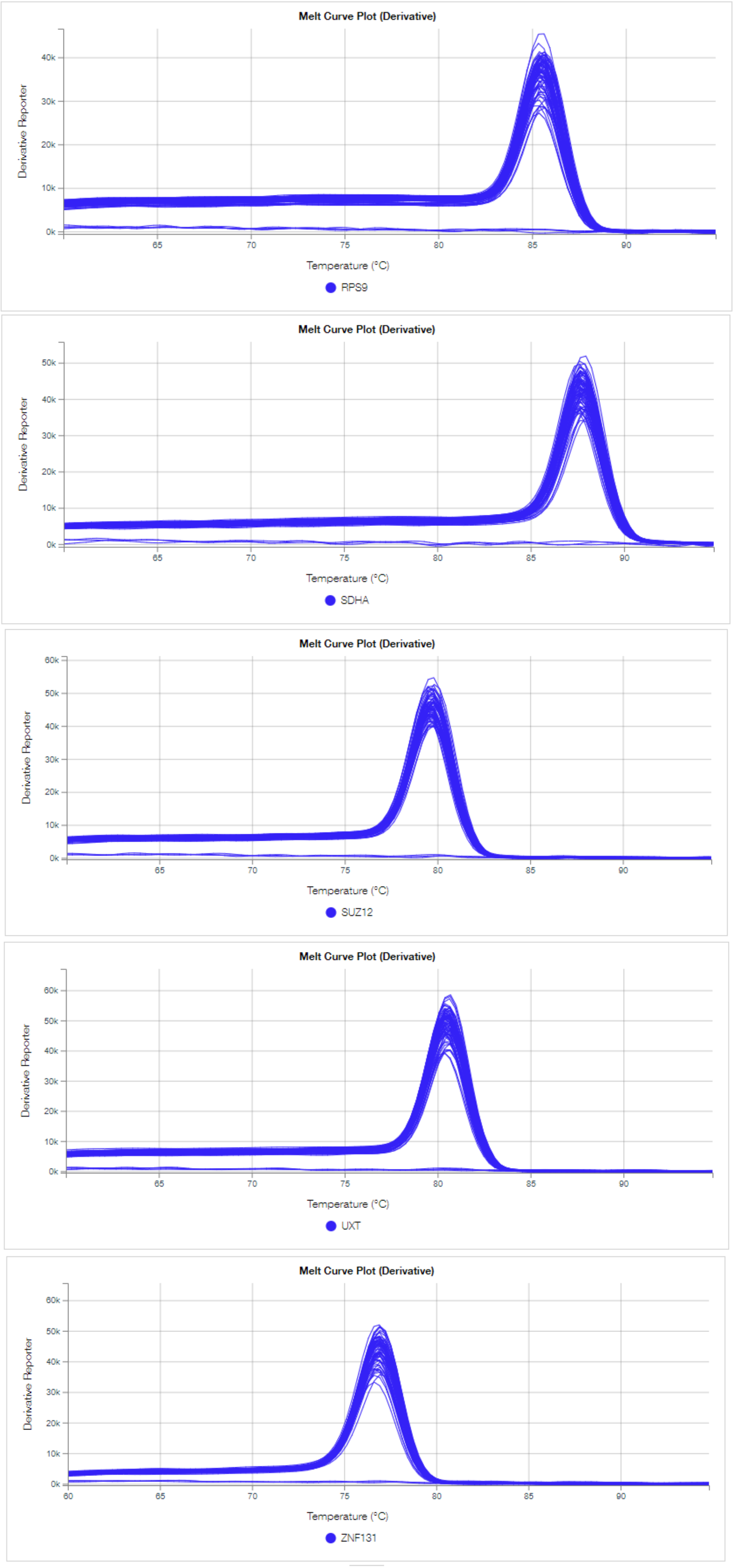


Figure S1. Dissociation curves obtained for tested reference genes. *ACTR1A* (actin-related protein 1A), *CNOT11* (CCR4-NOT transcription complex subunit 11), *HDAC1* (histone deacetylase 1), *HPRT1*

(hypoxanthine phosphoribosyltransferase 1), *RPL19* (ribosomal protein L19), *RPS9* (ribosomal protein S9), *SDHA* (succinate dehydrogenase complex flavoprotein subunit A), *SUZ12* (SUZ12 polycomb repressive complex 2 subunit), *UXT* (ubiquitously expressed prefoldin like chaperone) and *ZNF131* (zinc finger protein 131).

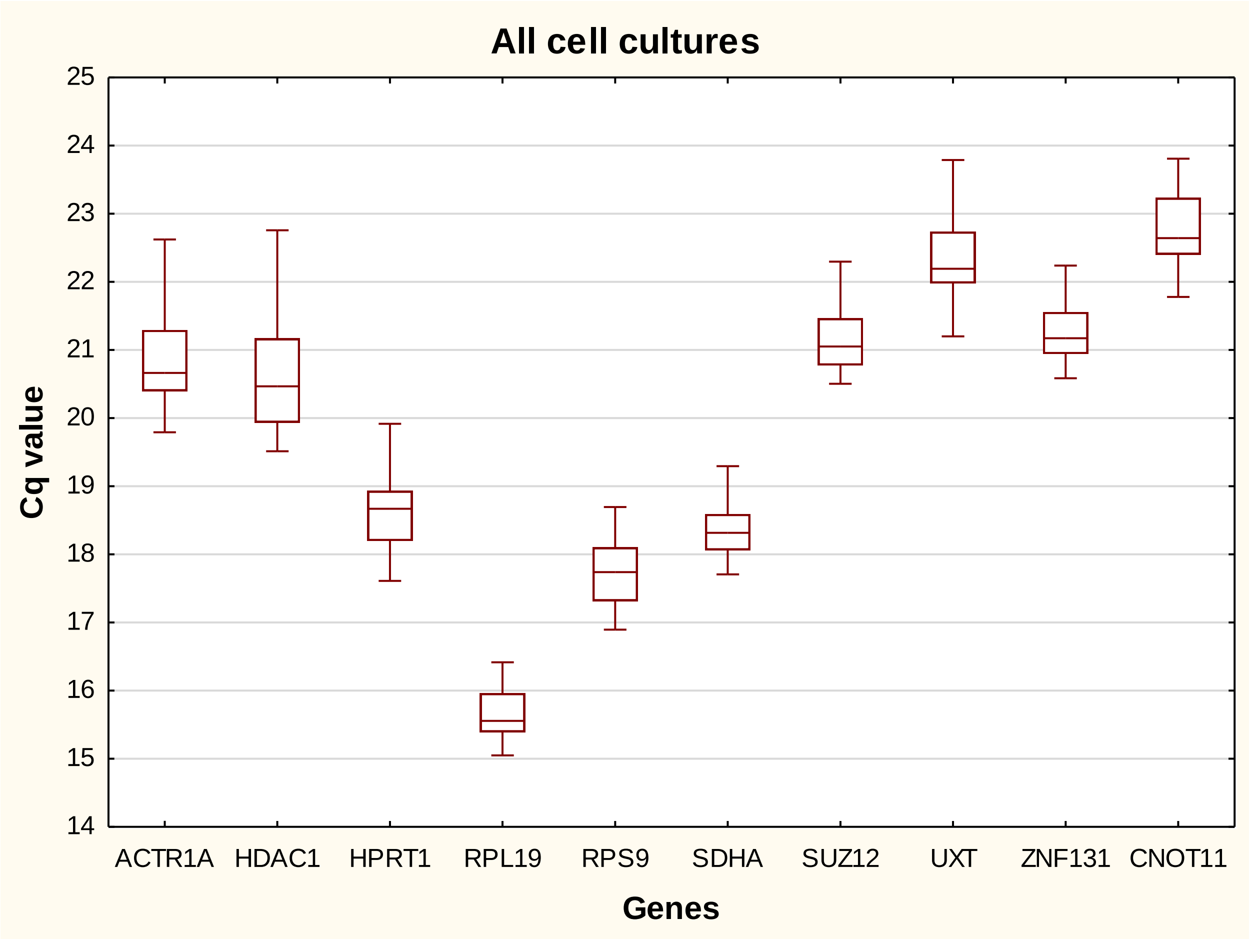


Figure S2. The quantification cycle values (Cq) for all examined genes. Data are shown in the box plots including the minimum and the maximum value (whiskers), the sample median (line in the middle), and the first and third quartiles (frame).

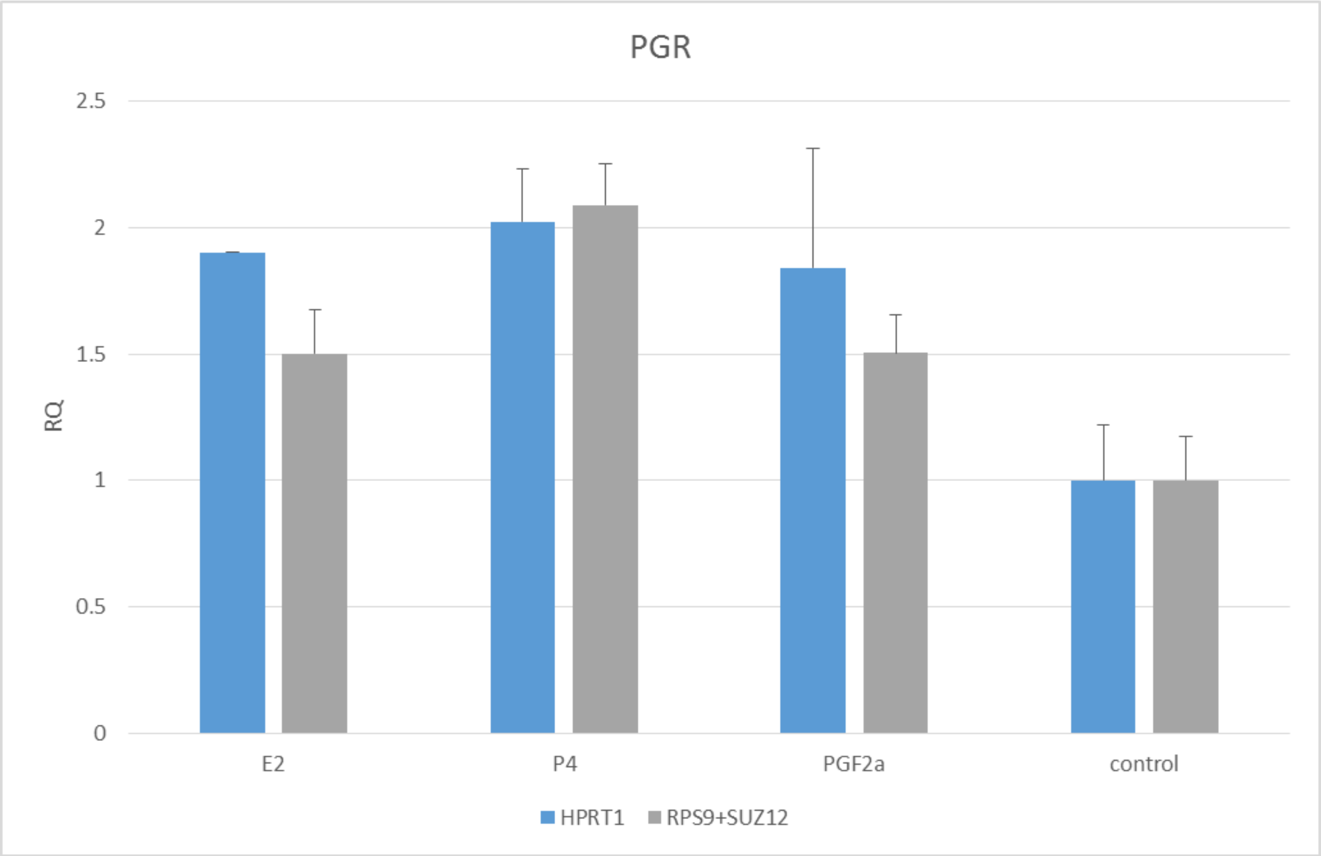


Figure S3. Relative expression of the *PGR* gene in the cell line obtained from the 4th month of pregnancy. Analysis was performed with normalization against two best performing RGs (*RPS9*+*SUZ12)* and the least stable RG, *HPRT1*. Data is shown as mean ± SD.

Table S1. Parameters derived from RT-qPCR analysis - slope, regression coefficient ($R^2$), amplification efficiency and melting temperature of the amplicon (Tm).

| Gene | Slope | $R^2$ | Efficiency (%) | Tm (°C) |
|---|---|---|---|---|
| *ACTR1A* | -3.19 | 0.998 | 105.663 | 84 |
| *CNOT11* | -3.20 | 0.999 | 105.223 | 87 |
| *HDAC1* | -3.29 | 0.998 | 101.384 | 80 |
| *HPRT1* | -3.49 | 0.993 | 93.288 | 78 |
| *RPL19* | -3.40 | 0.991 | 96.999 | 83 |
| *RPS9* | -3.16 | 0.998 | 107.060 | 86 |
| *SDHA* | -3.43 | 0.992 | 95.576 | 88 |
| *SUZ12* | -3.56 | 0.990 | 91.042 | 79 |
| *UXT* | -3.22 | 0.996 | 104.319 | 81 |
| *ZNF131* | -3.57 | 0.994 | 90.647 | 77 |
| *PGR* | -3.29 | 0.994 | 101.303 | 81 |
